# How to report professional practice in nursing? A scoping review

**DOI:** 10.1186/s12912-016-0154-6

**Published:** 2016-05-25

**Authors:** Marie-Eve Poitras, Maud-Christine Chouinard, Martin Fortin, Frances Gallagher

**Affiliations:** Faculté de médecine et des sciences de la santé, Université de Sherbrooke, Québec, Canada; Département des sciences de la santé, Université du Québec à Chicoutimi, Québec, Canada; Centre intégré universitaire de santé et de services sociaux du Saguenay-Lac-Saint-Jean, Hôpital de Chicoutimi, 305, Saint-Vallier, Chicoutimi, G7H 5H6 Québec Canada

**Keywords:** Activity, Domain, Nursing, Professional practice, Role, Scoping review, Structure

## Abstract

**Background:**

Nursing professional practice in different contexts of care has been widely described in evidence-based literature. Currently, there is no consensus on a common structure for these descriptions. Understanding and comparing similar practices is made difficult by the varying nature of descriptions provided in scientific literature. Purpose of the study: 1) to report research methods found in the scientific literature that were used to describe the practice of different health professionals; 2) to report on the main concepts used to describe the practice of these health professionals; 3) to propose a structure for the description of the practice in nursing.

**Methods:**

A scoping review following a five-stage approach: 1) identifying the research question; 2) identifying relevant studies; 3) selecting studies; 4) charting data; 5) reporting results. The Medline, CINAHL, psychARTICLES, psyCRITIQUES, psycEXTRA, Psychology and Behavioral Science Collection and psycINFO databases were searched. Each study was analyzed and extracted data were classified by categories and structures used to describe the health professional practices.

**Results:**

Forty-nine studies were included. In these studies, quantitative, qualitative or mixed methods were used to describe professional practice in different health disciplines. Three major concepts were reported most frequently in describing professional practice: roles, domains and activities. The concepts varied greatly among authors. We found that to define roles or to characterize a professional practice, activities must be described and organized on the basis of different domains.

**Conclusions:**

A promising structure for describing nursing professional practice is proposed by the authors of this review. The structure facilitates the accurate description of all domains and activities performed by nurses in different contexts of practice, and will contribute to the development of knowledge about nursing practice in different contexts based on shared concepts.

**Electronic supplementary material:**

The online version of this article (doi:10.1186/s12912-016-0154-6) contains supplementary material, which is available to authorized users.

## Background

Nursing professional practice defines the exercise of the profession [1, 2]. It is an amalgam of practice standards, nursing skills and expected professional performance [3]. Professional practice sets the base for nursing exercise at the conceptual level and allows nurses to ensure quality of care among different contexts of care [4, 5]. Nursing professional practice has been widely described at the conceptual level, both in the scientific literature and by the bodies that regulate the nursing profession [1, 3, 6]. Though essential, these descriptions use different concepts and a heterogeneous vocabulary, preventing a clear understanding of nursing professional practice. For example, to present the role of nurses in advanced practice, Gardner et al. use the professional literature and the legislative framework of the profession [7], while Reuter-Rice [8] explains the professional practice of nurse practitioners within a specific context: pediatric practice [8]. While summarily guiding nursing professional practice, the current literature fails to provide real clarification on what nurses actually do in clinical practice [9].

To date, several studies have focused on describing nursing professional practice in specific contexts [2]. For example, Halcomb et al. [10] used a national survey to describe the demographics, clinical roles and competencies of primary care nurses through results presented by clinical activities and domains of practice. A similar structure was also used by Lukewich et al., who used a cross-sectional survey [11] to describe the practice of primary care nurses in chronic disease management. Other authors, such as Phillips et al. [12], preferred a qualitative design to describe how nurses manage obesity. Results are presented by role. Very few of these authors base their description of practices, roles or activities on a frame of reference. They do not provide a definition of a “role.” It is up to readers to piece together their own idea of professional practice from the results they come across. Often misapprehended, the heterogeneous concepts and vocabulary used to describe nursing professional practice in various contexts make it hard to draw comparisons or identify similarities between them. There are no guidelines or consensus on how to provide a good description of nurses’ practice. Other healthcare disciplines, such as occupational therapy, physiotherapy and medicine, are also doing research to describe professional practices and improve knowledge. We also observed a certain degree of variability in the concepts used in these disciplines. The one exception is Bent et al. in their study on the practice of occupational therapists, which presents a framework offering a structure to map professional practice by fields and tasks [13]. Thanks to its structure and components, their framework offers a promising perspective for describing the practices of other health professionals.

At this stage of the development of nursing knowledge, it is essential to map out the existing literature to summarize how professional practice has been described. This knowledge could be enhanced by existing knowledge from other health disciplines that also care for patients. This common structure could then be used to describe professional practices, enabling a better representation of them and facilitating knowledge sharing in the nursing profession and nursing sciences.

The aim of this scoping review was: 1) to report on research methods found in the scientific literature that were used to describe health professional practices; 2) to report the main concepts used to describe professional practice in various health care disciplines; 3) to propose a structure for the description of professional practice in nursing. Hopefully, this finding will be used in another project to describe primary care nurses’ practice with patients with chronic diseases.

## Method

### Design

We used the scoping review approach to examine the scientific literature and to describe the main concepts used to describe professional practice in various health care disciplines. This method was chosen because it contributes to the mapping of key concepts underpinning a research area and the main sources and types of evidence available [14]. We preferred a scoping review to a systematic review for the following reasons: 1) a systematic review would typically focus on a well-defined question with a limited range of appropriate study designs, whereas a scoping review can address broader topics and a broader range of study designs; 2) a systematic review seeks the answers to questions addressed by a subset of studies whose quality is assessed, whereas a scoping review is unlikely to address specific research questions, and consequently, unlikely to assess the quality of the studies reviewed [15].

The review was inspired by Arksey and O’Malley’s [15] five-stage approach and the principles of knowledge synthesis established by Grimshaw [14] : 1) Identifying the research question; 2) Identifying relevant results; 3) Selecting studies; 4) Charting data; and 5) Reporting results.

### Stage 1: Identifying the research question

- What methodologies are used to describe professional practice in health care-related scientific literature?
- What concepts are used to describe professional practice in health care-related scientific literature?

### Stage 2: Identifying relevant studies

A review of the literature in specific health disciplines was conducted. For a comprehensive view of professional practice descriptions, health disciplines other than nursing were also searched. Our research strategy (keywords, MESH, databases) was developed by the authors of the scoping review, as they have significant and relevant experience in synthetic methodology and literature review. Occupational therapy, physical therapy and medicine were the three disciplines chosen, due to the similarity of practical contexts (providing direct care to patients) and populations in terms of professional practice. Within those disciplines, any context of practice was considered, to provide a better understanding and description of what is found in the health literature. The databases of Medline, CINAHL, psychARTICLES, psyCRITIQUES, psycEXTRA, Psychology and Behavioral Science Collection and psycINFO were searched. Keywords used were: practice analysis, role delineation, professional practice, role, nurse, nursing, occupational therapy, physical therapy, medicine, physician, mixed methods, qualitative descriptive study and quantitative descriptive study. A search strategy using Medical Subject Headings (MESH) and text words was developed for each keyword and database. In view of the changing nature of professional practices, the 2003–2014 time span was chosen, highlighting contemporary descriptions anchored in the past ten years. We limited our search to studies published in English or French. See Additional file 1 for more detailed information on the database search strategy.

Only studies meeting the following criteria were included in the scoping review: 1) Quantitative, qualitative or mixed-method research used to describe the professional practice; 2) Health disciplines: nursing, occupational therapy, physical therapy and medicine and 3) Any context of practice. Editorials, commentaries, case reviews, study replication or study which did not present content about how the authors describe the professional practice were excluded.

### Stage 3: Selecting studies

**Fig. 1:**
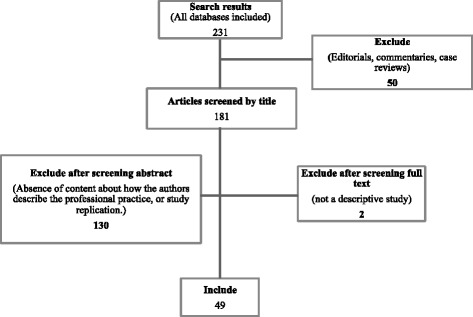
Flow diagram

The search query identified 231 articles. After reviewing the titles, abstracts and admissibility criteria, a total of 49 papers met all inclusion criteria and were kept for the review (see Fig. 1). A summary of the eligible studies is provided in Table 1.

**Table 1:** Summary of studies included (*N* = 49), categorized by health discipline and key concepts measured

Author(s)	Country	Design	Participants (*n*)	Key concept measured									
				Role	Domain	Activity	Task	Intervention	Ability	Knowledge	Skills	Responsibility	Attitude
Nursing (*n* = 31)													
Baghi et al. (2007) [21]	US	Practice analysis	Ambulatory care nurses (*n* = 499)						X	X			
Berger et al. (2010) [53]	US	Practice analysis	Nurses caring for morbidly obese and bariatric surgical patients (*n* = 1084)				X						
Bevans et al. (2011) [20]	US	Practice analysis	Clinical research nurses (*n* = 412)	X	X	X							
Brown et al. (2012)[51]	US	Practice analysis	Oncology nurse navigators (*n* = 330)	X			X						
Clark et al. (2009) [17]	US	Practice analysis	Advanced practice nurses in hospice and palliative care (*n* = 180)				X						
Feltovich et al. (2010) [56]	US	Practice analysis	Nurses working in infection prevention (*n* = 3771)		X		X						
Garbin et al. (2013) [19]	US	Practice analysis	Nephrology registered nurses (*n* = 940)			X							
Gardner et al. (2013) [7]	Australia	Practice analysis	Advanced practice nurses (*n* = 660)	X	X	X							
Long et al. (2013) [52]	Australia and New Zealand	Practice analysis	Paediatric intensive care unit nurse educators (*n* = 15)			X			X	X	X		
Muckle et al. (2009) [63]	US	Practice analysis	Nurse anesthetists (*n* = 3805)							X			
Muckle et al. (2012) [46]	US	Practice analysis	Nurse anesthetists (*n* = 9003)		X								
Ortelli et al. (2006) [47]	US	Practice analysis	Academic nurse educators (*n* = 2217)		X		X						
Pellino et al. (2003) [62]	US	Practice analysis	Pain Management orthopaedic nurses (*n* = 41)	X				X					
Ramirez et al. (2006a; 2006b) [22, 23]	US	Practice analysis	Nurse practitioners providing care in the emergency department (*n* = 582)		X		X						
Reuter-Rice (2013) [8]	US	Practice analysis	Acute care pediatric nurse practitioners (*n* = 291)	X			X						
Rice et al. (2007) [57]	US	Practice analysis	Advanced practice psychiatric and mental health nurses (*n* = 28)				X						
Roberts et al. (2013) [58]	US	Practice analysis	Orthopaedic nurses (*n* = 1194)							X			
Shuriquie et al. (2008) [48]	Jordan	Practice analysis	Medical-surgical staff nurses and practical nurses (*n*= 348)	X	X	X					X		
Strasser et al. (2006) [18]	US and Canada	Practice analysis	Occupational health nurses (*n* = 5586)	X			X		X	X			
Webb et al. (2008) [55]	US	Practice analysis	Legal nurse consultants (*n* = 369)	X	X		X			X			
Willens et al. (2010) [50]	US	Practice analysis	Pain management nurses (*n* = 585)		X		X						
Bottorff et al. (2005) [26]	Canada	Qualitative description	Genetic nurses (*n* = 22)	X	X							X	
Hopkins et al. (2012) [29]	England	Qualitative description	Epilepsy specialist nurses (*n* = 19)	X	X	X							
Poghosyan et al. (2012) [33]	Armenia	Qualitative description	Nurses (*n* = 43), staff (*n* = 29) and head nurses (*n* = 14)	X									
Susilo et al. (2013) [37]	Indonesia	Qualitative description	Nurses in informed consent (*n* = 27)	X					X	X		X	
Kucera et al. (2010) [31]	Australia	Narrative account	Nurses (*n* = 142 story)	X		X							
Salmela et al. (2012) [34]	Finland	Phenomenology	Nurse leaders (*n*= 17)	X			X						
Stewart et al. (2013) [36]	UK	Phenomenology	Critical care nurses (*n* = 5)	X		X				X			
Hunter et al. (2010) [43]	Australia	Mixed methods	Nurses working with older people in long term care: (registered nurses (*n* = 48) and nurse managers (*n* = 16))	X		X			X			X	
Van Soeren et al. (2011) [42]	Canada	Mixed methods	Nurse practitioners in hospital setting (*n* = 46)		X								
Medicine (*n* = 6)													
Schuster et al. (2013) [59]	Japan and US	Practice analysis	Physicians in cardiovascular disease risk factor management (*n* = 48 Japanese and 53 US physicians)			X							
Ball et al. (2010) [24]	Australian and New Zealand	Qualitative description	General practice physician educators (*n* = 20)	X					X		X		X
Booij et al. (2013) [25]	Netherlands	Qualitative description	Physicians in treatment of Huntington’s disease (*n* = 15)	X									
Schoenborn et al. (2013) [35]	US	Qualitative description	Physicians during care transitions of older adults (*n* = 18 physicians, 11 home healthcare administrative and field staff, 4 social workers, 3 nurse practitioners, 3 physician assistants, and 1 hospital case manager)	X			X						
Johansen et al. (2010) [30]	Norway	Narrative account	Physicians providing cancer care	X			X			X			
Kee et al. (2012) [39]	Australia	Qualitative description	Direct observation of emergency department consultations (*n* = 130 h)	X		X							
Occupational therapy (*n* = 4)													
Bent et al. (2005) [13]	US	Practice analysis	Occupational therapists (*n*= 2675)		X		X			X			
Dalton et al. (2013) [27]	Canada	Qualitative description	Occupational therapists caring for children with physical disabilities	X			X						
Habib et al. (2013) [28]	Bangladesh	Qualitative description	Occupational therapists and disaster management (*n* = 6)	X		X							
Turpin et al. (2012) [38]	Australia	Qualitative description	Occupational therapists (*n*= 462)	X	X	X							
Physical therapy (*n* = 7)													
Dimick et al. (2009) [44]	United States, Canada, Australia and New Zealand	Practice analysis	Physical therapists providing hand therapy (*n* = 760)			X							
Dockter et al. (2008) [75]	US	Practice analysis	Advanced practitioners in women’s health physical therapy (*n* = 176)							X			
Donato et al. (2004) [64]	US	Practice analysis	Physical therapists practising in primary contact care settings (*n*= 119)							X			
Gorman et al. (2010) [61]	US	Practice analysis	Acute care physical therapists (*n* = 254)	X	X					X			
Perry et al. (2008) [54]	US	Practice analysis	Neurologic physical therapists (*n* = 187)			X	X		X	X			
Swisher et al. (2008) [49]	US	Practice analysis	Cardiovascular and pulmonary physical therapists (*n* = 89)	X		X	X						
Masley et al. (2011) [32]	US	Grounded theory	Physical therapists working in acute care (*n* = 18)	X									
Multidisciplinary (*n* = 1)													
Curchoe et al. (2008) [45]	US, Saudi Arabia and Canada	Practice analysis	Health professionals working in infection prevention (*n* = 3771, 825 nurses)	X			X						

### Quality appraisal

As with all scoping exercises, the aim was to map the current literature with regard to the description of professional practice in the four selected health disciplines, rather than to assess the quality of the particular studies chosen [15, 16]. As suggested by reference authors Arksey & O’Malley [15] and Grimshaw [14] , a quality appraisal of included literature is not required when performing a scoping review. To be included in our study, the only quality criterion a paper had to satisfy was to have been peer-reviewed.

### Stage 4: Charting data

The 49 papers retained for the scoping review were read and summarized by MEP. The research team generated a template for data extraction. MEP analyzed each paper and did the data extraction according to Arksey & O’Malley’s [15] recommendations. Synthesis and interpretation of the extracted data were done to highlight the methodologies and structures used to describe professional practices [15]. Data were classified by author, year of publication, study location, study population, context of care, study aim, methodology, structure and concepts used to describe the professional practice. MEP examined which concepts related to professional practice were presented in the results and how they were organized. For each paper, the logic of the structure of the description was extracted based on the terms used by the authors and the description of practices provided (e.g., role, activity, domain, intervention, task). Structures used were analyzed, interpreted and summarized. Methodologies were also classified by category and sub-category. For each paper, the co-authors discussed the methodology and structure used for the description of the practice, until a consensus was reached. MF, FG, and MCC participated in validating the papers and analyzing the methods and structures extracted from them. They also played a mentoring/coaching role throughout the process.

## Results

The Results section represents Stage 5 of the scoping review approach, namely reporting results. The latter are presented in two parts.

### Research methods used to describe professional practice quantitative methodologies (*n* = 31)

Quantitative descriptive studies were found useful in the literature. Such studies examine responses obtained through self-report questionnaires in population surveys and are defined as practice analysis or role delineation studies.

### Practice analysis or role delineation (*n* = 31)

The terms “practice analysis” and “role delineation” appear in studies with similar goals in the literature [8, 17, 18]. They are used to signify that one does more than simply analyze a task [18]. We observed that papers using “role delineation” focused on differences in practice between two complementary professions [19] or between professionals with different levels of experience [20], while studies using “practice analysis” drew a portrait of practice in general [17, 21]. In view of the objectives of this scoping review, the term “practice analysis” was preferred.

A common approach emerged in cross-sectional surveys conducted for studies using practice analysis. It can be summed up in eight steps: 1) develop a panel of experts; 2) determine the demographic variables of the population of interest; 3) identify which practice element to describe (e.g., roles, tasks, activities) with the help of a panel of experts and the literature; 4) classify tasks or activities according to the domains identified; 5) determine the measurement scale; 6) develop the questionnaire; 7) pre-test; and 8) conduct the survey. The steps used vary slightly from one study to another. The statistical methods used included descriptive or regression analysis [7], variance analysis [19] and factor analysis [22, 23].

### Qualitative methodologies (*n* = 16)

All the qualitative studies examined sought to describe or explore professional practice [24–39]. For example, Bottorff et al. [26] set out to depict the roles of nurses involved in genetic clinical services for adults; they used semi-structured interviews with 22 nurses across five Canadian provinces. Other authors used a phenomenological approach [34, 36], grounded theory [32] or narrative [31].

Data collection methods varied from one study to another. Some used telephone interviews with open-ended questions [24, 26, 32], while others used face-to-face individual interviews [25, 28, 30, 32, 35, 37], focus groups [29, 33, 36, 37], narrative [31], observation [39] or self-administered questionnaires [38]. Sometimes multiple data sources were used [33, 37].

Data analysis was similar in selected studies. Thematic analysis was either inductive [24, 29, 30, 32, 33, 38] or relative to a framework [13, 34, 36, 40]. Researcher triangulation was also done in certain studies [24, 29, 32, 35–38], as was analysis validation with participants [32, 33, 37]. Finally, for all the studies examined, data were presented by theme or category, supported by verbatim [25, 27, 28, 32, 37], examples [31, 36] or figures [34, 36].

### Mixed methods (*n* = 2)

Two studies were identified that used mixed methodologies [41]. Both were sequential, one exploratory [42] and one converging [43].

Hunter and Levett-Jones [43] used a combination of questionnaires, individual interviews and an examination of documents explaining roles. Van Soeren et al. [42] did the same, but began with a qualitative component (individual interviews and focus groups) describing and classifying the activities of nurse practitioners. As for data analysis, Hunter and Levett-Jones [43] integrated both types of data for the analysis and presentation of results. Van Soeren et al. [42] used quantitative data to support data collection in the qualitative phase. For qualitative data, content analysis was used based on a theoretical model. Quantitative analyses were descriptive and done so as to compare groups of activities [42, 43].

### Main concepts: conceptualizing and operationalizing professional practice

As seen in Table 1, several key concepts were used to describe professional practice in health disciplines. Ten studies used a more conceptual definition of professional practice [7, 31, 44–50], while others were more operational [7, 8, 13, 17–20, 22, 23, 44–60]. As an example, Bevans et al. [20] define clinical research nurses’ professional practice in five specific domains of activities : 1) Study Management, Care Coordination and Continuity, Contributing to the Science and Human Subjects Protection. Those domains allow knowing the specific activities realized by nurses. In the same way, Clark et al. [17] give a list of more than 100 activities to describe what nurses in palliative care do. At the opposite, Hopkins and Irvine [29], describe professional practice of epilepsy specialist nurses with four core values: 1) holistic care; 2) Time for patient; 3) Continuity of care and 4) Proactive/responsive. After reading these core values, it is hard for the reader to clearly imagine what the nurses do exactly with patient.

The three most relevant concepts for nursing practice (and those most used by nursing organizations)—role, domain and activity—form the basis of a promising structure for the description of professional practice. Table 2 presents the concepts we retained, their definition as understood by the authors of this scoping review, and examples from a clinical primary care context.

**Table 2:** Concepts, definitions and examples related to nursing professional practice

Concepts	Definitions	Examples
Role	Function assumed by the nurse, modulated by professional norms, a legislative framework, a scope of practice and a social system.	Primary care nurses for patients with chronic conditions
Domain	Set of activities of the same nature requiring specific knowledge and expertise.	Patient care management
Activity	Actions undertaken by the nurse to help a patient go from a current state of health to the one described in expected results.	- Providing nursing care - Referring the patient to community resources - Performing intervention plan - Communicating with the patient’s healthcare providers

### Professional role

Thirty studies examined sought to describe professional practice in terms of the role of professionals in a given context [7, 8, 18, 20, 21, 24–39, 43, 45, 48, 49, 51, 55, 58, 61, 62]. However, very few studies elaborated on what a role is, conceptually [7, 20, 31, 45, 49, 58, 62]. They focused on describing a role in terms of how it is defined in professional literature and the legislative framework [7, 58, 62]. In quantitative and mixed-method studies, role is not measured per se. It emerges as a theme through the description of activities, tasks or interventions. It is a perceived role, rather than a role defined by a professional practice standard.

### Domain

To describe the role of professionals, fifteen authors determined the domains in which they practise [7, 13, 22, 23, 26, 29, 38, 42, 47, 48, 50, 55, 56, 61, 63]. The activities that comprise a practice can be found by first establishing the domains in which a professional practises [13]. For example, Bent et al. [13] identified five domains in which occupational therapists practise their profession, then set out their multiple tasks in the different domains. In the literature review, we noticed that for the most part domains are not evaluated directly. They are used to explain scope of practice, but also to group the tasks, activities or interventions comprising a professional practice.

### Activity

The concept of activity is central to the description of a professional practice. Nearly all authors, except for Ball et al. [24], Habib et al. [28], Hopkins & Irvine [29], Masley et al. [32] and Stewart et al. [36], describe professional roles or practices through a census of activities. *Activities* [20, 44, 47, 49, 56, 59, 62] also imply *interventions* [58, 64] and *tasks* [23, 30, 33, 34, 37–39, 47]. The authors mentioned do not seem to differentiate among the three concepts (activity, intervention, task), nor do any provide a conceptual definition of the term they use.

### Structure of professional practice

**Fig. 2:**
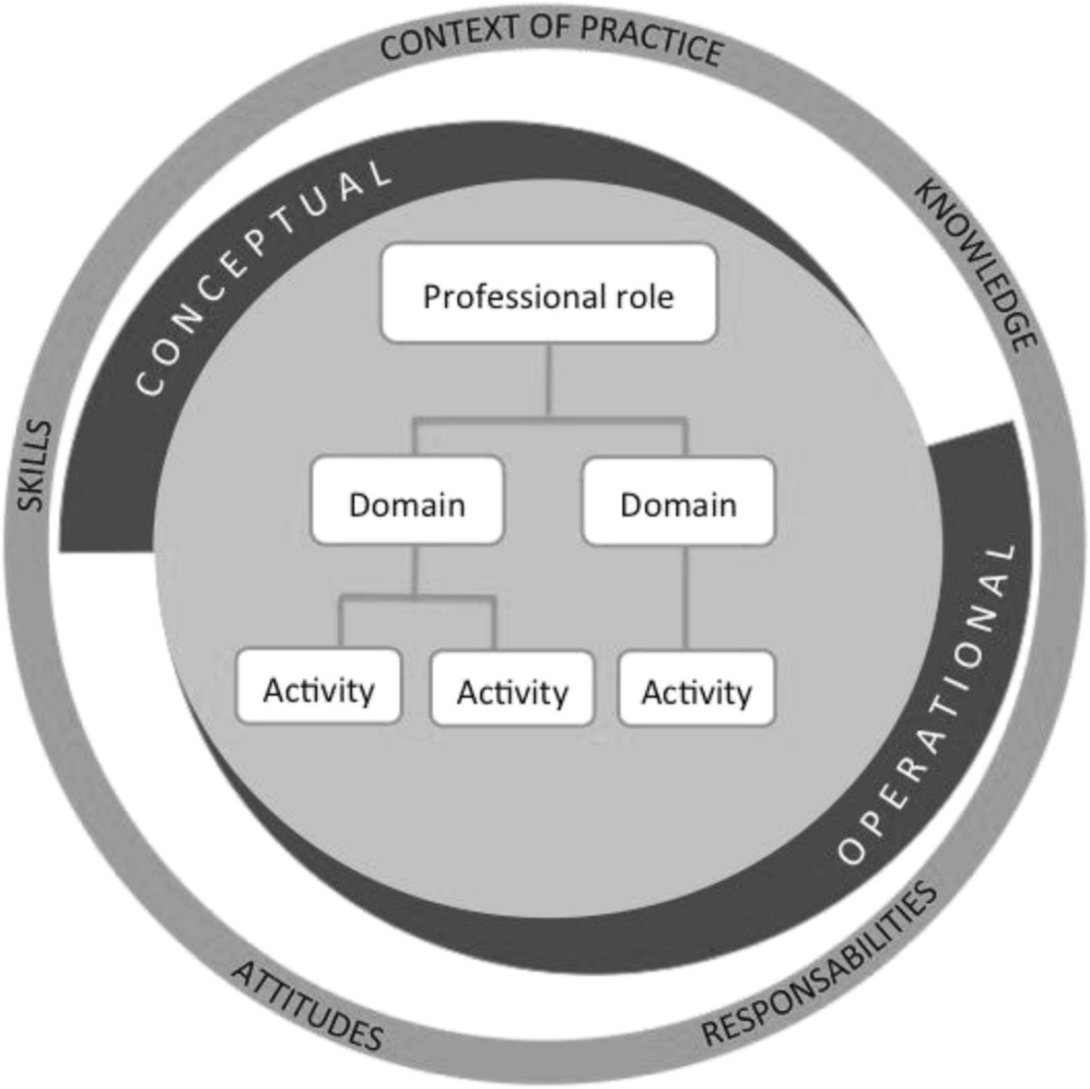
Structure for the description of professional practice in nursing (adapted from Bent et al.) [13]

From our analysis of the literature, a pattern emerges when describing professional practice. This pattern is presented in Fig. 2. Most authors refer to the concepts of role, domain and activity. However, use of those concepts in describing professional practice varies greatly among authors but not necessarily among health discipline. Though activities are clearly identified, the concepts of roles and professional practice are poorly defined. There is confusion in the literature between the concepts of role, activity, intervention and task.

## Discussion

### Methodologies used

This scoping review examined different ways of capturing professional practice through research using quantitative, qualitative and mixed methods. The results show consistency in how practice is described across various studies in specific health domains.

Practice analysis is an approach that uses cross-sectional surveys in a specific population of professionals. This implies the study of either roles, knowledge, behaviours, skills, activities and tasks, or the interventions of this group of professionals [51, 52, 61]. This methodology is commonly used by bodies that grant licences to practise [21, 44, 53–55, 61, 63]. The practice analysis perspective provides a way to describe current large-scale practices. The descriptive data so generated can contribute to the development of a theoretical practice framework [13, 21]. However, sampling methods as well as low response rates may limit the generalization of results [18] and can lead to under- or over-reporting certain activities [65]. Finally, the greatest challenge of practice analysis is to find strategies to ensure an acceptable response rate [66].

In contrast, using qualitative methodology to describe professional practice allows an in-depth description of a practice, in a natural clinical setting, from the perspective of professionals. The use of various data sources enhances the description and improves the validity of the results presented. In some cases, the qualitative data generated enable the construction or validation of a model describing professional practice [29, 31, 32, 34–37]. Qualitative methodology provides a more inductive description of professional practice. However, the results make it difficult to implement recommendations for practice as a whole, because the concepts used are specific to a unique context of practice.

Mixed-method design brings comprehensiveness, depth and richness to the description of professional practice [41]. The qualitative and quantitative data generated by the different phases are complementary. Quantitative descriptions provide an overall view of practice by describing professional activities in terms of proportion and frequency. Qualitative descriptions provide depth and highlight the various points of view of the actors concerned. However, conducting mixed-method research can be time-consuming, and tends to require multiple financial and professional resources [41].

Depending on their specific objective, it is up to researchers to choose the method that is best suited to their research question. The mixed-method design seems an interesting approach that contributes richness and complementarity to the description.

### Main concepts and structure used to describe professional practice

We found that how the concept of role is envisioned can be compared to the conclusions of Biddle [67], Biddle and Thomas [68], Meleis [69], Roch and Ouellet [70] and O’Rourke [5]. Biddle and Thomas [68] argued that, conceptually, a role is a function taken or assumed by a person, supported by an ensemble of norms that define what the behaviour of that person must be in that position [67, 68]. This approach is also taken by Meleis [69], who adds that a role is created by knowledge linked to the profession and by interactions among actors in a social system. Both explanations are along the same lines as Roch and Ouellet [70] and O’Rourke [5], who explain that a role is a function modulated by the legislative framework of the profession, the practice area, training, specificities of the patients cared for, and organizational expectations in the practice setting [5, 70]. Such a role can be defined by the activities assumed by one person, modulated by professional norms, a legislative framework, a scope of practice and a social system. That is the definition we have chosen to use.

We found that to define roles or to characterize a professional practice, activities must be described and organized on the basis of different domains [7, 8, 13, 18, 20, 26, 34, 44, 49, 50, 55, 56, 62]. A domain represents the scope of a professional’s role [26]. For nurses, the domain of practice generally consists of evaluating health condition, determining and ensuring nursing care and treatment plan, and providing nursing and medical care and treatment in order to maintain and restore the health of a human being [4].

Creating a domain allows us to group activities. However, it seems apparent that the concepts of activity and task coincide with the concept of intervention reported by Gordon [71], Sidani & Braden [72] and Burns & Grove [73]. Specifically, Gordon [71] explains that an intervention is an action undertaken by the nurse to help a patient go from a current state of health to the one desired [71]. Sidani & Braden [72] add that an intervention refers to a treatment, therapy, procedure or action carried out by a professional for a client in a specific situation. Finally, Burns & Grove [73] add that this can be an activity. In light of the literature reviewed and the theoretical currents cited, we conclude that the concepts of activity, intervention and task are synonymous. The concept of activity is used from here on.

This hierarchical representation of professional practice extracted from our analysis of the studies is similar to that presented by the National Board for Certification in Occupational Therapy [74] and used by Bent et al. [13]. The latter propose a model on which to structure professional practice [13]. This model, adapted to the findings of the present review, is particularly appropriate for the nursing profession. It suggests a general structure for future descriptions of nursing practice, regardless of context of practice. This implies that professional practice should be defined by domains with which activities are associated. Bent et al. [13] also suggest that knowledge should also be part of this structure, since it is required for the completion of activities in each domain. Our study allowed us to improve on the model proposed by Bent et al. [13] by recognizing the conceptual nature of the notions of role and domain, while also showing that what gets measured in a clinical setting are activities. Bent et al. [13] recognized the crucial presence of specific knowledge for each activity. In addition to knowledge, it have been found in literature that skills, attitudes, responsibilities and context of practice all have an impact on the performance of activities and on the quality of that performance [18, 21, 44, 51–55, 58, 61, 63, 64, 75]. These elements enrich the description of practice and were added to the model proposed by Bent et al. [13] as seen in Fig. 2.

The most important contribution of this review for the nursing profession is a comprehensive and innovative way to describe professional practice. Nursing practice covers a large number of roles, domains and activities. This vast range of possibilities poses a significant barrier to developing a comprehensive description of the reality of nursing practice. The evidence-based, hierarchical structure proposed will advance the description of professional practice and contribute knowledge based on similar concepts. This will help in comparing, evaluating and reporting domains and activities in different contexts of care in comparable countries. The ultimate outcome could be a uniform comprehension of nursing expertise and its emerging contribution to health care.

### Strengths and limitations

This scoping review allowed us to report and better understand different ways of describing professional practice in the health-related literature through the concepts of roles, domains and activities. Despite rigorous analysis and the synthesis process used, certain limitations must be considered. First, the selection and analysis of articles were done by the primary author only; however, the selection criteria were respected rigorously and analysis was done systematically following Arksey & O’Malley’s five-stage approach [15]. Also, the process described in this paper is sufficiently transparent to allow the research to be replicated. Second, the quality of the articles examined was not evaluated, save for the “peer-reviewed” criterion. However, the main objective of this review was to report methodologies and concepts used to describe professional practice, not to conduct a synthesis of their results, as would be required in a systematic review [76]. Also, certain health disciplines were not included in the literature review. Nonetheless, given the number of papers included and the similarities in the descriptions of practice, a certain consistency in language is apparent. Most of the studies examined were from the field of nursing. This indicates a common structure in the nursing literature that points toward a certain consensus.

## Conclusion

In nursing, as in other health professions, professional practice is a complex concept linking practice standards, performance standards and deontological values [3, 5, 70]. In all the disciplines, study authors claim to be describing a professional practice, but rarely do they do so thoroughly. Describing activities grouped into various domains does not equate to describing a professional practice, but only one or another of its components. Compared to other professions, the scope of practice in nursing is large and multifaceted [3, 6]. The structure presented in this scoping review will enable the accurate description of all domains in which nurses practise along with the activities they perform. Future descriptions should consider the conceptual and operational definition of roles. To provide an accurate picture of the complexity and richness of nursing expertise, activities performed must be clearly identified and integrated into practice domains. To ensure understanding of the descriptions provided, it is essential to provide a faithful depiction of the context of nursing practice. Ideally, any description of practice must be supported by a clear schematic representation of the author’s perception of professional practice.

## Additional file

Additional file 1: Detailed search strategy. (DOCX 18 kb)
