# Supplementary material for: How to report professional practice in nursing? A scoping review
Source: BMC Nurs. 2016 May 25;15:31. doi: 10.1186/s12912-016-0154-6 (PMC4880971; doi:10.1186/s12912-016-0154-6)
Supplement: Additional file 1: — Detailed search strategy. (DOCX 18 kb) [file 12912_2016_154_MOESM1_ESM.docx]

Databases : Medline, CINAHL, psychARTICLES, psyCRITIQUES, psycEXTRA, Psychology and Behavioral Science Collection and psycINFO

Search conducted December 8, 2013

1. practice analysis

2. “practice analysis”

3. nurse

4. occupational therapist

5. physiotherapy

6. medicine

7. 1 or 2

8. 7 and 3

9. 7 and 4

10. 7 and 5

11. 7 and 6

13. role delineation

14. “role delineation”

15. 13 or 14

16. 15 and 3

17. 15 and 4

18. 15 and 5

19. 15 and 6

20. mixed method

21. qualitative study

22. quantitative study

23. role

24. professional practice

25. 20 and 23 or 24

26. 21 and 23 or 24

27. 22 and 23 or 24

28. 16 or 17 or 18 or 19

29. 8 or 9 or 10 or 11

30. 25 or 26 or 27 or 28 or 29

31. 30 and 3 or 4 or 5 or 6

32. limit year = 2003-current

33. french or english language

Number of results: 231
